# The influence of the environment on the patient-centered therapeutic relationship in physical therapy: a qualitative study

**DOI:** 10.1186/s13690-023-01064-9

**Published:** 2023-05-17

**Authors:** Jaume Morera-Balaguer, Mª Carmen Martínez-González, Sonia Río-Medina, Víctor Zamora-Conesa, Marina Leal-Clavel, José Martín Botella-Rico, Raquel Leirós-Rodríguez, Óscar Rodríguez-Nogueira

**Affiliations:** 1 Nursing and Physical Therapy Department, Health Sciences Faculty, CEU-Cardenal Herrera University, CEU Universities, Elche, 03201 Spain; 2 SALBIS Research Group, Nursing and Physical Therapy Department, Faculty of Health Sciences, University of León, Astorga Ave. 15, Ponferrada, 24401 Spain

**Keywords:** Patient centered care, Physical therapy, Professional-patient relations, Therapeutic relationship, Environment

## Abstract

**Background:**

Currently, in the scientific literature there is a great interest on the study of strategies to implement patient-centered care. One of the main tools for this is the therapeutic relationship. Some studies suggest that the perception of the environment in which the treatment takes place can influence the perception of its quality, but this is not explored in physical therapy. For all these reasons, the aim of this study was to understand the influence of the environment in which physical therapy treatment takes place on the patients’ perception of the quality of the patient-centered therapeutic relationship in public health centers in Spain.

**Methods:**

A qualitative study analysed thematically using a modified grounded theory approach. Data collection used semistructured interviewing during focus groups.

**Results:**

We conducted four focus groups. The size of the focus groups ranged from six to nine participants. In total, 31 patients participated in these focus groups. Participants described a series of specific experiences and perceptions relating to the environment, which they felt were influential in the establishment of therapeutic patient-centered relationships, including six physical factors (Architectural barriers, Furniture, Use of the computer, Physical space, Ambiet conditions, and Privacy) and six organizational factors (Patient-physical therapist ratio, Treatment interruptions, Social factors, Continuity with the professional, Lack of professional autonomy, and Coordination or communication among team members).

**Conclusion:**

The results of this study highlight environmental factors that affect the quality of the therapeutic patient-centered relationship in physical therapy from the patient’s point of view, and emphasize the need for physical therapists and administrators to underline the need to review these factors and take them into account in their service delivery.

## Background

Currently there is a great interest in studying strategies to implement patient-centered care (PCC) in all areas of healthcare [1–6]. According to Morgan and Yoder [7], PCC is a holistic (bio-psycho-social-spiritual) approach to delivering care that is respectful and individualized, allowing negotiation of care, and offering choice through a therapeutic relationship where persons are empowered to participate in health decisions at whatever level is desired. This model has been recommended by several prestigious professional organizations, such as the Institute of Medicine and others [8–11] as a way to increase the quality of health care.

One of the most relevant factors for the establishment of PCC is the quality of the therapeutic relationship, or the relationship that is established between the care providers and their patients [5–11]. There is growing consensus that the quality of care depends directly on communication and the relationship between patient and therapist, both in the field of physical therapy and in other health disciplines [3, 4, 12–16].

Several authors have demonstrated the relationship between aspects of the environment in which the service is provided the therapeutic relationship [17–19].

Furthermore, the importance of the environment is being widely studied in marketing and environmental psychology. These two disciplines recognize that certain socio-environmental factors can influence the perceptions of service quality. Marketing literature recognizes the relevance of three specific service attributes of the physical environment: design, environmental conditions (nonvisual aspects such as temperature, humidity, ambient sound, as well as visual aspects such as lightening) and social factors (the number, type and behaviors of people evident in the service setting) [20]. Environmental psychology include elements of service organization [21]. From the perspective of environmental psychology, there is a broad line of research on the influence of these factors on variables such as environmental stress, environmental overload and deprivation, psychophysiological and behavioral effects, and performance [22].

In rehabilitation settings, the environment in which the treatment is carried out has not been sufficiently studied. Some authors have carried out studies that relate the environment with patient satisfaction or perceived quality [23, 24]. Nonetheless, limited research has been conducted on the possible influence of the environment where treatment is administered and the perception of the quality of the therapeutic relationship between patient and professional [17–19, 25].

This article explores the influence of the environment in rehabilitation settings on the therapeutic relationship in Spain. The spaces in which the service took place were a general treatment room, in the presence of other patients, generally with a wide variety of pathologies, with shared material resources, and even a shared therapist. Programs typically last for multiple weeks, with 1 session per day.

This research is part of a wider study attempting to perform an in-depth analysis of the perceptions and experiences of patients and physical therapists that influence the perceived quality of the patient-centered therapeutic relationship in physical therapy.

This study aimed to understand the influence of the environment in which physical therapy treatment takes place on the patients’ perception of the quality of the patient-centered therapeutic relationship in public health centers in Spain. To this end, this study explores the experiences and perceptions of patients regarding this aspect.

## Methods

### Study design and sample

A qualitative study was conducted based on the use of focus groups, analysed thematically using a modified grounded theory approach, which is inductive in nature, to allow themes of importance to patients related to the Influence of the Environment on the Patient-Centered Therapeutic Relationship [26].

Qualitative methods usually highlight human experiences as emotions, expectations and attitudes [27]. We therefore felt that this was the best type of research design to gain insight into the perceptions and experiences of our participants. We used focus groups because group interactions can trigger responses and build insights that may not arise during individual interviews. Focus groups have been used previously to identify experiences and perceptions related to how health services are perceived [24, 25]. The study was approved by the Research Ethics Committee of the Cardenal Herrera CEU University and the Ethics and Research Committees of the General University Hospital of Elche, the Vinalopó Hospital, and the Provincial Hospital of Valencia. The authors followed the SQUIRE 2.0 checklist.

The inclusion criteria consisted of patients from physical therapy units in primary care and public hospitals in Spain with a minimum of 15 treatment sessions. The sole criterion for exclusion was the existence of any type of cognitive or communication disability.

Patients were recruited from three hospitals and six primary care centers. Purposive sampling strategy was used to include subjects with varying age, sex, and clinical conditions(28).This recruitment strategy is used to maximize the sampling heterogeneity, in order to identify and expand the range of variation or differences [28]. This enabled the selection of participants who could best provide insight into specific and personal experiences and perceptions regarding the issues being examined, rather than obtaining a representative sample, as would be sought in quantitative research [26]. The recruitment was carried out by the same physical therapists who were treating the patients, who volunteered after a meeting with a member of the research team, in which they explained the objectives of the project and their participation. Each physical therapist reviewed their list of patients to identify those who met the selection criteria, based on the medical history and their experience with the patient, drawing a list of possible participants.

The researchers made a selection of the profiles using intentional sampling to obtain a heterogeneous sample and to explore the phenomenon under study with sufficient breadth and depth [29]. An attempt was made to include subjects with different profiles considering the sociodemographic characteristics that in previous studies have been shown to influence perceptions of the quality of the therapeutic relationship [27]: patients from rural and urban areas, from hospitals and primary care centers, of different ages, sex and pathologies. All members of the research team work as university professors and had no relationship with the participants in the study.

**Fig. 1:**
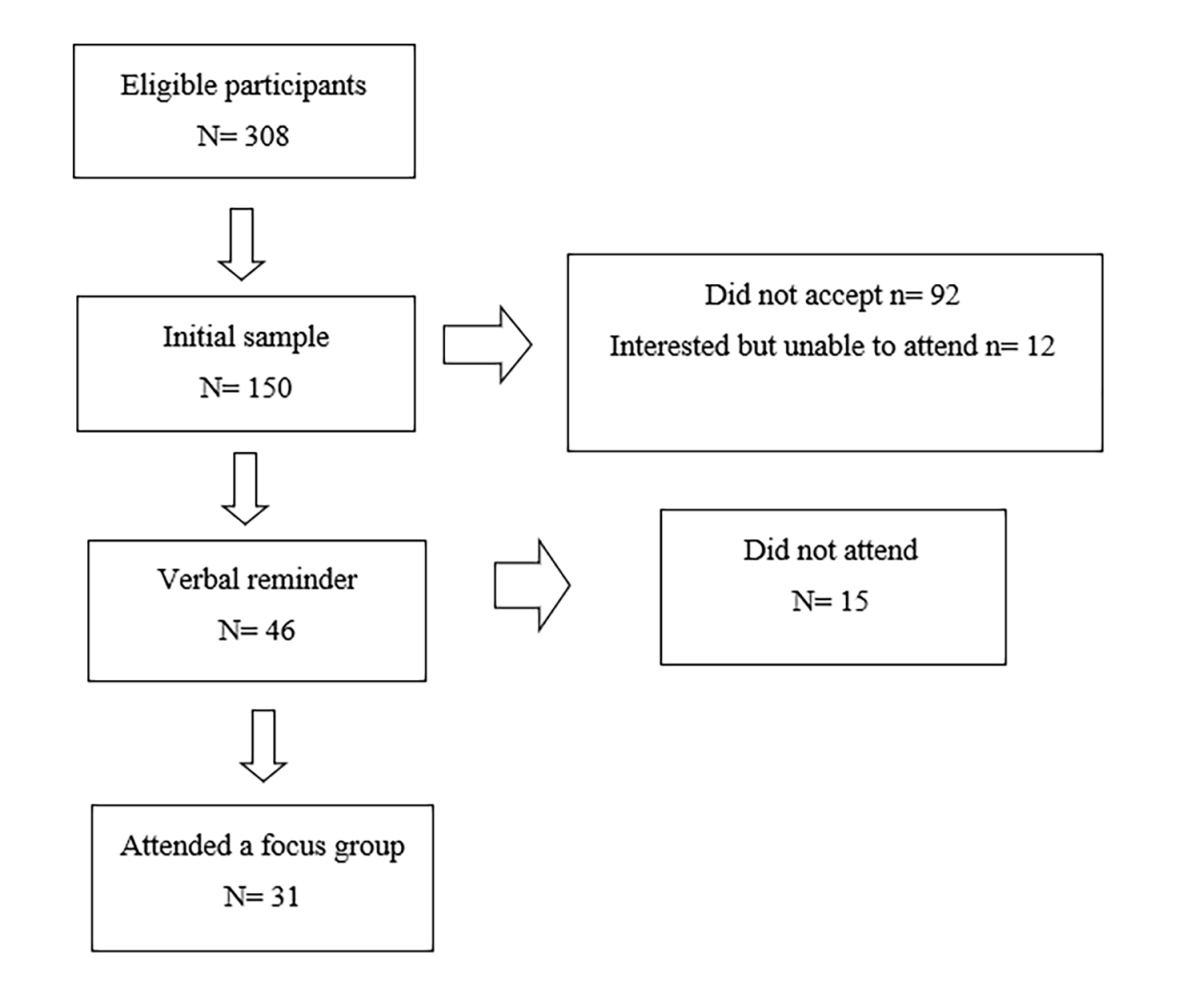
Stages of selection process for focus groups

Although the research team took into account that the size of the final sample would depend on the saturation of the information, established as the point at which no new information was extracted from the focus groups, we initially selected 150 subjects, which would allow us to form between 4 and 11 focus groups (it is generally considered that an adequate group size being between four and 10 or 12 participants, with the optimal size being between six and eight individuals so that subgroup are not formed) [30]. Figure 1 presents a scheme of the stages of selection process.

### Data collection

All focus groups were led by a moderator (J. M.-B.) and an assistant (Ó. R.-N.). All sessions were held in a room that was separate from the care center (community centers run by associations and institutions) with the aim of creating an appropriate environment for discussion, away from the clinical context of physical therapy care units. An ad hoc topic guide with predetermined questions was created and used based on a literature review (Table 1). Group discussions were audio and video recorded. Focus groups were held until data saturation was achieved, meaning that no new categories emerged after analysis of the previous focus group data [31]. The data collection process took place in a room adjoining the rehabilitation room with enough privacy to be able to carry out the session without interruptions. It was carried out between the months of May and October, always at a time that allowed all possible participants to participate, when the Physiotherapy sessions had already finished, between 7:00 p.m. and 8:00 p.m. Each focus group met once.

**Table 1:** Patient focus group questions guide

1. What do you most value about the physical therapist?
2. What did you like the most about your relationship with the physical therapist?
2.1 In which situations have you felt most comfortable with the physical therapist?
2.2. In which situations have you felt uncomfortable with the physical therapist?
2.3. Did the physical therapist appear the way he/she genuinely is? (the physical therapist was able to show his/her limitations, recognize errors, be natural…)
3. Which attitudes or behaviors of the physical therapist make you trust him/her?
4. When do you tend to talk with the physical therapist?
5. What do you tend to talk to the physical therapist about?
6. Have you felt understood and supported during treatment?
7. Do you believe the physical therapist sought your collaboration during treatment?
7.1 In order to establish objectives
7.2. In order to establish the means for achieving these objectives
8. Do you believe the physical therapist gives you all the information you need to know?
9. Do you tend to understand the physical therapist? Yes, no; when? and why?
10. How do you describe the environment in which your relationship with the physical therapist takes place? And, how does this environment affect you?
11. What aspects do you consider are worth improving in the relationship between the physical therapist and yourself?

### Data analysis

The sessions were transcribed verbatim (V. Z.-C. and S. R.-M.) for independent analysis. The names of the participants were anonymized using a numerical code assigned for the transcripts and quotations. A modified grounded theory approach was used for data analysis [26, 32], incorporating data collection, coding, and analysis, and using a process of constant comparison without the component of developing a theory in light of the results [33].

Three authors (J. M.-B., Ó. R.-N. and M. C. M.-R.) independently reviewed the transcripts and coded sentences containing significant units of analysis. These were grouped into categories using a combination of emerging codes. The same three authors reviewed and compared their findings to reach agreement on codes and categories. Three rounds of coding and discussion were conducted with the aim of improving the reliability of the coding process and developing clearer categories. This process was iterative with the collection of data from subsequent transcripts. No new categories emerged at the end of the fourth focus group, which implied that saturation had been reached.

The next level of analysis sought to identify the relationships between categories and the grouping of categories with uniformity into themes and sub-themes with a higher conceptual level. To do so, the similarities and contrast within the data were compared by the investigators and data that seemed to cluster together were sorted into different levels. To evaluate the consistency of the final themes and subthemes, two researchers (S. R.-M. and V. Z.-C.) verified their agreements based on a blind review using codes for the same passages from two transcripts [33]. Any disagreement between the two researchers was resolved by discussion. At each step, an independent author (M. C. M.-R.) played the role of reviewer to verify whether the analysis was systematically supported by the data, with the intention of enhancing dependability, transferability and confirmability [26].

## Results

**Fig. 2:**
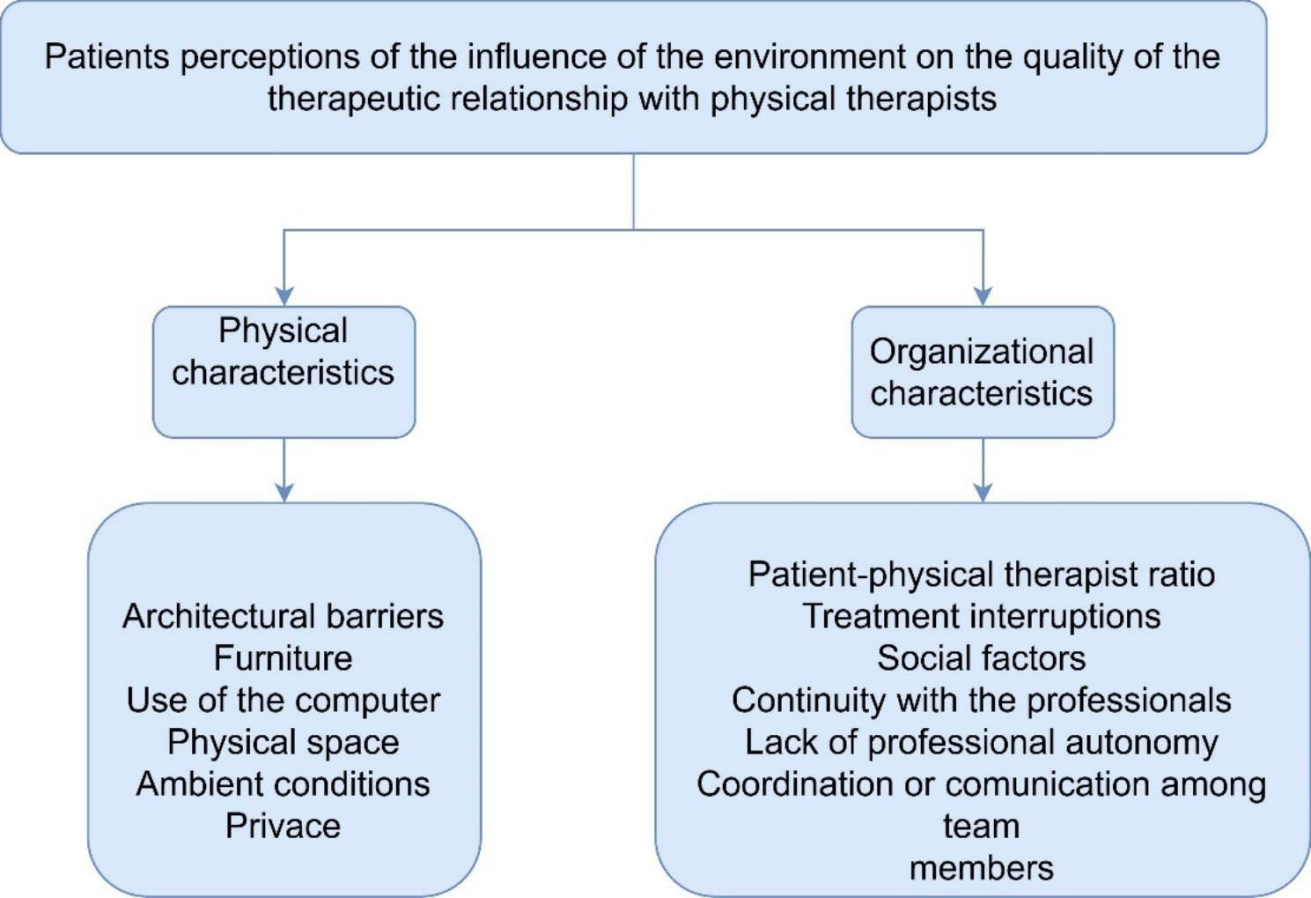
Outline of themes, subthemes and categories

Four focus groups were held until information saturation was reached. The size of the focus groups ranged from six to nine participants. In total, 31 patients participated in these focus groups. The characteristics of the participating patients are shown in Table 2. The analysis of these focus groups identified that the participants’ experiences and perceptions was related to one of the following themes: physical characteristics, and organizational characteristics. Figure 2 shows the final summary of themes and subthemes, following a hierarchy that was defined by the level of abstraction.

**Table 2:** Sample characteristics

**Gender** [data provided: n (percentage)]	
Woman	21 (67.7)
Man	10 (32.3)
**Level of studies** [data provided: n (percentage)]	
Primary	19 (61.3)
Secondary	10 (32.3)
University	2 (6.5)
**Pathology** [data provided: n (percentage)]	
Musculoskeletal	21 (67.7)
Neurological	10 (32.3)
**Age (years)** [data provided: mean ± standard deviation]	52.7 ± 14.9
**Treatment time (weeks)** [data provided: mean ± standard deviation]	27 ± 76.9

In the text below, the themes and sub-themes are presented, accompanied by quotes from the participants.

### Physical characteristics

The physical characteristics are those characteristics of the physical space in which the therapy takes place that influenced the perception of the Patient-Centered Therapeutic Relationship from the participants’ point of view. In our study, we found six physical factors.

*(a) Architectural barriers*: Many participants discussed the negative influence of “architectural barriers” in their relationship with their physical therapist. For example, the presence of columns in the treatment room may hinder visibility between the patient and physical therapist. Several patients also described the inconvenience of having the physical therapist’s office separate from the treatment room. The participants perceived that both aspects hinder the relationship between a patient and their physical therapist. For example, one patient stated:

*“Also, the architectural barriers that the room has because here is the rehabilitation room, a sink, a dressing room, the office and a room for electrotherapy. So of course, if they go into the office you can’t communicate with them…”* (Man, 48 years old, polytraumatized, 40 sessions).

*(b) Furniture*: Some patients told us that at times “unsuitable furniture” can make it difficult to establish a rapport of trust, and can also hamper communication between the physical therapist and the patient. For example, one patient spoke of the feeling of distrust caused by the existence of a table between the doctor and the patient, compared to the situation when she is in with the physical therapist:

*““He (Physical therapist) is next to you, or in front of you…there’s no barriers. But in other cases, there is a table in between, there is their chair, which goes up and down and you have your chair, you know, that gives you the feeling that you’re talking to the president of the company …you’re not at the same level, and and it is hard to build trust.”* (Woman, 68 years old, fibromyalgia, 42 sessions).

*(c) Use of the computer*: Similarly, several patients told us about the difficulty of establishing fluid communication due to the “use of the computer”, which causes a lack of fluidity in the verbal and non-verbal communication between both parties:

*“The treatment with her* (Physical therapist*), great, very kind, she worries about everything, tries to be on top of everything, but when she goes into the office she has to keep a file there on who has come, who hasn’t shown up…yes, the computer issue blocks them a lot….it makes communication difficult.”* (Woman, 27 years old, anterior cruciate ligament reconstruction, 20 sessions).

*(d) Physical space*: Several patients told us about the problems generated by a “physical space that was neglected, poorly maintained or too small” for the establishment of a good relationship with their physical therapist, as this could result in difficulty in establishing a relationship of trust with their physical therapist, because this could lead to a global perception of mistreatment that contaminates the patients’ perception of the professional providing treatment, even if they have no direct relationship with the space. According to one patient:

*“… and you see yourself in classrooms that measure 40 meters, where there are 20,000 machines, and often you have to do exercises that you can’t do…then the floor is very bad…the tile moves, there’s a cracked tile…you feel like you’re being treated very poorly.”* (Man, 63 years old, traumatic brain injury, 40 sessions).

*(e) Ambient conditions*: Some patients talked about the effect on their mood of some less tangible aspects of the space in which the treatment was performed, such as the temperature or the lighting, which can cause a passive state of mind in the patient, wich is not conductive to the relationship with the physical therapist.

*“The barracks they have for rehabilitation are a scandal. I was doing cardiac rehab. Apart from the fact that it was unbearably hot… it is third worldly, it doesn’t make you want to do anything.”* (Man, 58 years old. Heart attack, 20 sessions).

*“We’re in a kind of den where there are no windows, when the light is a little dim it seems like a storm is raging…the lighting is also important, and here it’s all kind of aseptic, it’s like a prison or something… It takes away the desire to do anything and to communicate with the physical therapist.”* (Woman, 45 years old, Colles’ fracture, 22 sessions).

*(f) Need for privacy*: Another factor commented on by many patients was the “need for privacy”. According to the participants, there is a lack of privacy in the spaces where the physical therapy treatment is carried out in public centers, which prevents patients from finding moments to talk to their physical therapist about delicate matters, making communication and the establishment of trust difficult. According to one of the participants:

*“…because if their doing something to you and it hurts, you want your moment, your space, and to be able to talk to the physical therapist…at that moment I would have said, “go on,“ but I would have cried because I wouldn’t have had people looking at me.”* (Woman, 73 years old, knee prosthesis, 30 sessions).

### Organizational characteristics

The organizational characteristics are those characteristics related to the organization of the service (interaction or interdependence among employees or providers and planning of material and human resources) that affect the patient-centered therapeutic relationship from the point of view of the participants. In our study we found six organizational factors.

*(a) Patient-physical therapist ratio*: Many patients complained that the “patient-physical therapist ratio was too high”. According to these patients, this became a problem for establishing a good relationship with their physical therapist because they had very limited time to work with them:

*“…he can’t attend each person individually, and the personal treatment with the physio is what you get over time because there are people who are going to do 10 sessions and practically don’t become acquainted with him.”* (Woman, 22 years old, fractured tibia and fibula, 25 sessions).

These time constraints meant that sometimes the physical therapist was unable to fully focus on the patient he/she was treating:

*“He is doing something to you but at the same time he is watching someone else, and so he is not 100% focused on what he is doing.”* (Man, 40 years old, shoulder surgery, 28 sessions).

*(b) Interruptions in the middle of treatment*: Another common complaint was “interruptions in the middle of treatment”. This was often due to the saturation caused by the patient-physical therapist ratio being too high, and the patients themselves understood this. However, this prevented effective and fluid communication, and the establishment of the necessary trust between the physical therapist and the patient:

*“It makes me a little stressed to know that she has very little time, as soon as she approaches you have to say “” me, me “”, and that doesn’t make you feel relaxed, I don’t think it’s professional, I know she’s professional but the conditions don’t make her professional. I think we all deserve individualized quality treatment, not having to beg for it.”* (Woman, 59 years old, low back pain, 18 sessions).

*(c) Social factors*: Social factors present in the treatment space, such as the number of patients or interactions between them, were relevant to the patients. This could determine the perception of a relaxed environment, conductive to relationships. For example, some participants told us that sharing space with others who were in the same situation or similar to their own encouraged them and made them more eager to come to treatment:

*“As for me, I liked the fact that people were close by, that they chatted and so on, because maybe you went there with a bad feeling, and the other person who was there also gave you a hand and so on.”* (Man, 78 years old, fibromyalgia, 40 sessions).

However, on some occasions, too many patients sharing the space simultaneously proved to be a problem for some participants, as they hindered patient privacy, caused delays in treatment and sometimes even caused excessive noise.

*“I’m happy to be able to share every day with others who are like me, I’ve even made friends here… but there are days when you don’t feel like it so much, because you’re more sensitive or whatever, and then you’d like it if there weren’t so many people, to be able to focus more on you and your physiotherapist.” (*Woman, 76 years old, 38 sessions).

*(d) Lack of continuity with the same professional*: Many patients explained that the “lack of continuity with the same professional” made it difficult to create a flow of communication, which hampered the establishment of a therapeutic relationship:

*“If that person has connected with you, and has given you positive vibes…and encourages you, and stimulates you and you function well, switching to someone else… you have to start again from scratch, and if you don’t connect so much, it’s like a colder environment in rehab.”* (Man, 60 years old, multiple sclerosis, 80 sessions).

*(e) Lack of professional autonomy*: Many participants perceived that the physical therapist had a “lack of professional autonomy”, which in turn had a negative influence on the establishment of trust towards the professional because they perceived that the physiotherapist did not answer them with total sincerity. According to one patient:

*“…because, often, the physio should work more individually and does not do so because the guidelines that the doctor has set are such and such. And the physio says “no, I can’t touch you because I’ve been told that I can’t do this. I should do it, but I can’t, since I have not been told to do it,” and in the end you don’t know who to trust.”* (Man, 52 years old, scapulohumeral periarthritis, 38 sessions).

*(f) Lack of coordination or communication among team members*: Another frequent comment among participants was the perception of a “lack of coordination or communication among team members”, which caused patients to end up distrusting the people who treated them. One patient reported:

*“…when it comes to the person who has worked with me, great, the big problem is the contact between the doctor who sends you, the physical therapist, and the patient, that’s the problem.”* (Woman, 46 years old, fibromyalgia, 30 sessions).

Some patients also perceived contradictory information at times between professionals of the rehabilitation team and other departments, such as traumatology, which made them lose their confidence in the professionals and in the system in general:

*“The physio told me that he was going to give me another treatment, and he told me that if it doesn’t get better, they will have to operate on me, and the traumatologist said both times I went in that they would either infiltrate or operate.”* (Woman, 48 years old, supraspinatus tendinitis, 20 sessions).

## Discussion

The objective of this study was to understand the influence of the environment in which physical therapy treatment takes place on the patients’ perception of the quality of the patient-centered therapeutic relationship in public health centers in Spain. The results of this study show that the patient’s perception of the quality of the therapeutic relationship established with their physical therapist depends, in part, on their experiences and perceptions with the physical and organizational environment of the institution where they receive physical therapy treatment.

These results are in line with previous studies that relate patients’ experiences and perceptions with the environment and other constructs such as perceived quality [24, 34–39], or patient satisfaction [34–42] in health care services, or even in any service [20]. This suggests that there is a direct relationship between these constructs and the therapeutic relationship, as other authors have already partially demonstrated [34]. We believe it would be interesting to explore this mutual influence.

In addition, through our findings we have deepened our understanding of the environment factors that influence the perception of the Patient-Centered Therapeutic Relationship in Physical Therapy from the patient’s point of view.

Our study adds two interesting topics related to Facility design that we’re not found in other studies: the influence of furniture, which can hamper communication between the physical therapist and the patient, and the use of computer, which can lead to a lack of fluid communication. We consider these findings important as they can become both barriers and facilitators of a good therapeutic relationship.

Some of our participants’ experiences and perceptions related to organizational characteristics are directly related to the perceived quality of relationship with their physiotherapists. Ours findings support previous studies that relate these factors to professional-patient interaction [17–19] or to patient satisfaction [43].

Our participants stated that an excessive patient-physical therapist ratio, and interruptions in the middle of the treatment (which was often caused by the ratio itself) hampered their relationship with the physical therapist. Harrison et al. (36) found that patients were dissatisfied when they noticed that their physical therapists seemed rushed, which they interpreted as a lack of interest in the patient. Some authors stated that it influences the patient’s perception of quality (24,37,38) Other authors have found that patients need more time to discuss certain aspects of their treatment with their physical therapists that they feel unsure of (39). This demonstrates the importance of establishing joint decision making, one of the cornerstones of the PCC, to have enough time for each patient. These findings are consistent with our study and demonstrate the importance of these environmental characteristics for the establishment of the patient-centered therapeutic relationship.

Our study demonstrates the importance of continuity of care by a single professional. Patients consider this continuity to be a necessary factor in creating a relationship of trust with the physical therapist, which is necessary for the establishment of a good patient-centered therapeutic relationship [16, 44, 45]. Beattie et al. (39), in a study conducted with 1502 patients, found that 71.2% of those who stated they were fully satisfied with the treatment once it was completed had been treated by a single physical therapist. Subsequently, Medina-Mirapeix et al. [40] obtained similar results. These findings are in line with our study.

The participants suggested that lack of coordination or communication among team members sparks a sense of distrust of the patient towards the professional, which in turn makes it difficult to stablish the patient-centered therapeutic relationship. Scholl et al. [46] carried out a literature search to examine what dimensions make up the concept of PCC. These included the integration of medical and non-medical care, coordination and continuity of care, teamwork and teambuilding. These findings coincide with our study, showing that many of the characteristics of the environment that influence the establishment of a therapeutic relationship of quality are also dimensions of the concept of PCC. This is unsurprising if we consider that the concept of PCC focuses on the characteristics of the relationship between patient and professional as one of the fundamental strategies for its implementation.

We have found that the perception of lack of professional autonomy of the physical therapist was important for the establishment of a quality patient-centered therapeutic relationship, because it creates mistrust in the patient regarding his physiotherapist. We have not found this result in any of the studies reviewed. However, we believe that it is a topic that would be worth delving into, to find out the extent to which this affects both the therapeutic relationship and other health indicators.

This study presents several limitations. The retrospective nature of this study may mean that participants’ perceptions and experiences have suffered from memory bias. However, the fact that the data was collected simultaneously from several participants, and that, in most cases, several of them were treated by the same physical therapist and at the same center, we believe minimizes this potential problem. Furthermore, the nature of the data collection method (focus groups) may have caused a bias in the form of emotional contagion among participants. However, the fact that the moderator was sufficiently trained to avoid this situation, and the method of analysis, including three independent researchers, were able to mitigate this bias. Finally, given the sample size and the participation of patients, only from some public health centers of the Community of Valencia, caution should be taken when generalizing results.

### Implications for research

Given the current interest in the patient-centered health care model, and the scant literature that relates the environment in which it is carried out with said model, and taking into account that the results of our study cannot be widespread, we believe that future research could focus on expanding the study sample to increase the likelihood of generalising its results.

## Conclusions

The physical and organizational characteristics of the environment in which patients receive physical therapy treatment influence their perception of the quality of the therapeutic relationship. These findings add to the existing knowledge relating environmental factors with the perception of quality of the patient-centered therapeutic relationship, and highlight the need for physical therapists and managers to review the environmental factors that may be influencing the relationship from the patient’s point of view. This is especially important at a time when patient-centered care is being increasingly implemented. In the future, it would be interesting to study the influence of the environment in private centers, as well as in the treatment of different pathologies, such as neurological or pediatric patients.

The results of this study can be used by managers of centers where physical therapy care is provided to improve aspects that may be creating barriers to the establishment of a patient-centered therapeutic relationship. This may in turn improve many of the relevant outcomes of these centers, such as perceived quality or patient satisfaction. Physical therapists can also benefit from these results by understanding the environmental characteristics that can influence these results and, thus, explain to patients the reality of the center from a more genuine point of view, thus increasing patients’ trust, and, consequently, strengthening adherence to treatment and joint decision making.

## Data Availability

The dataset used and analyzed during the current study are available from the corresponding author.

## List of abbreviations

PCC: patient-centered care
